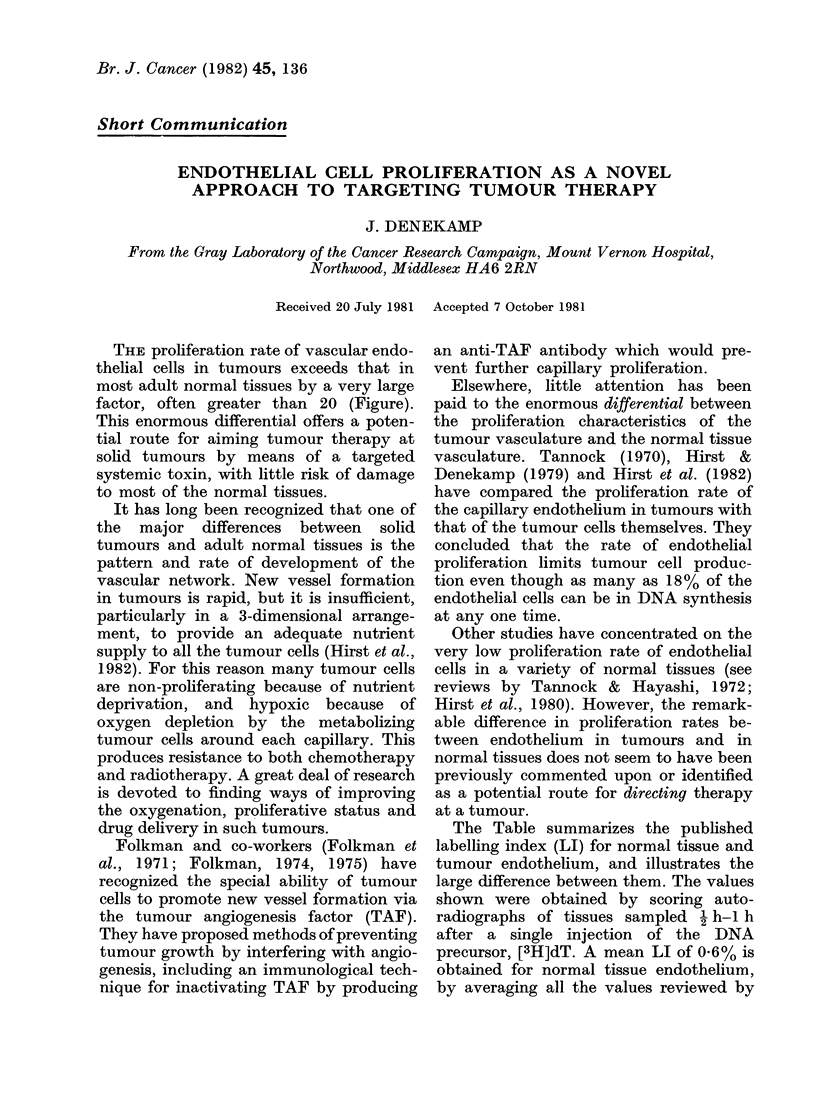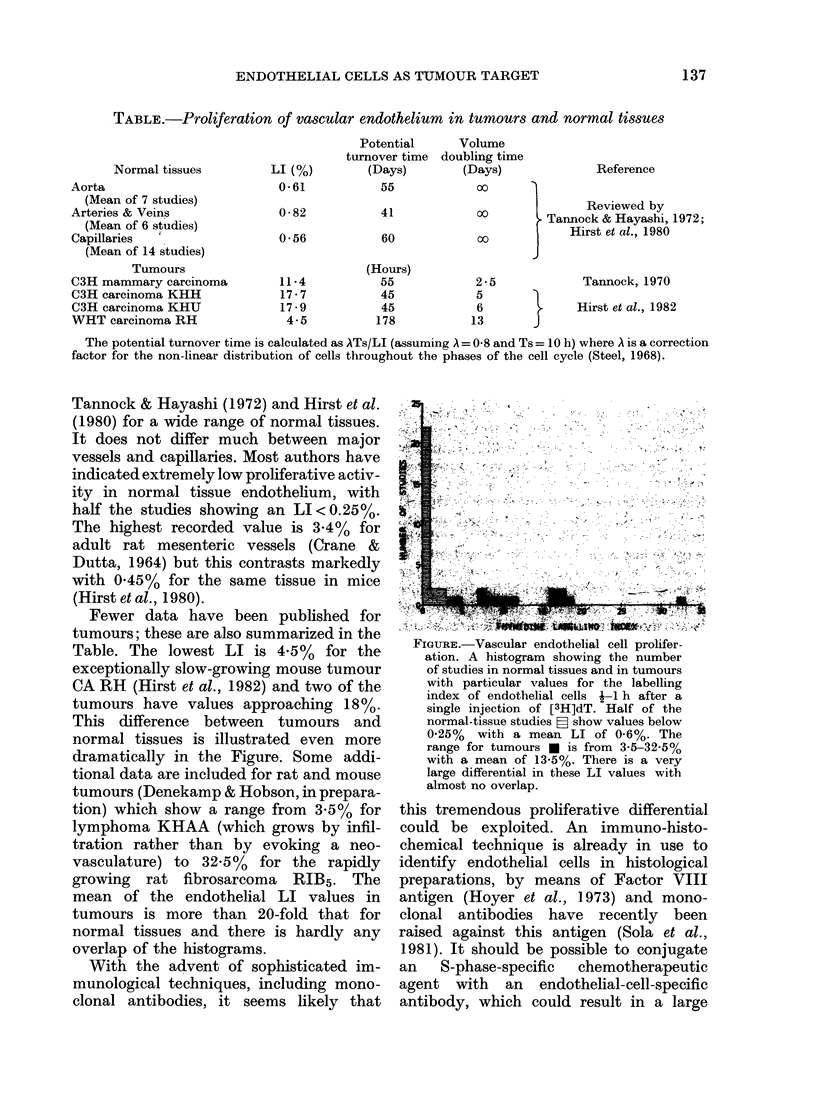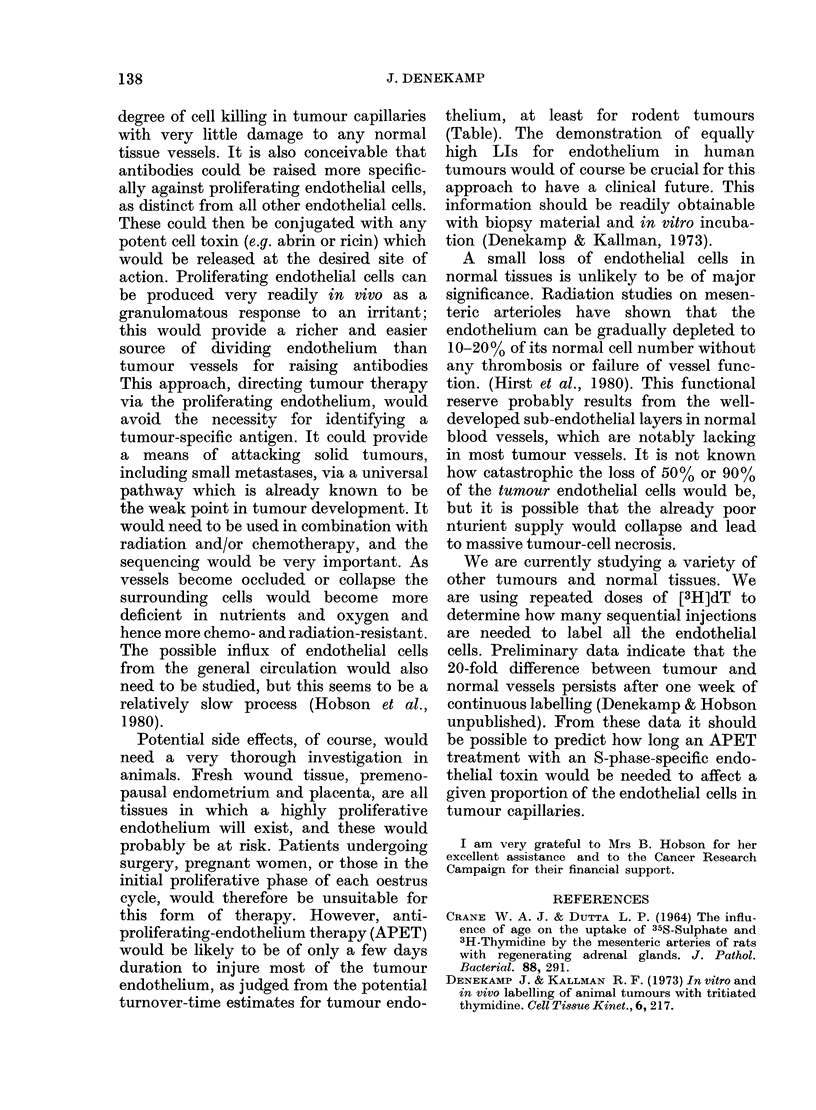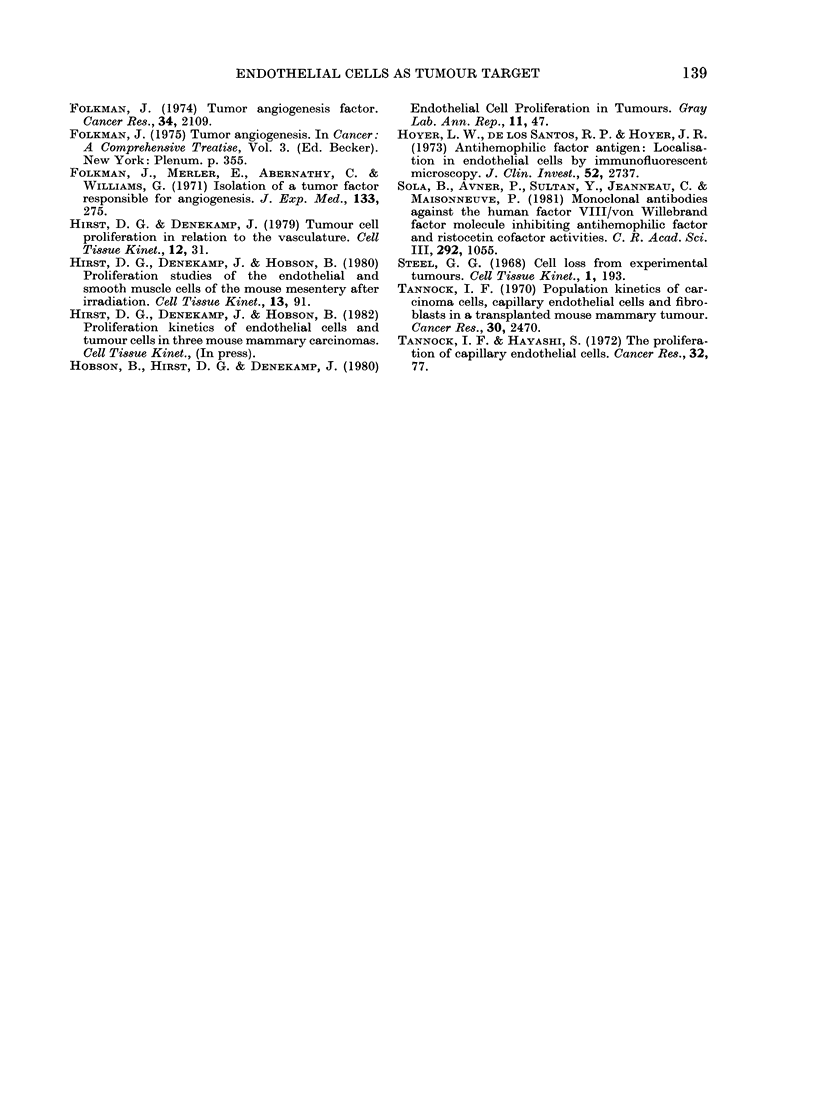# Endothelial cell proliferation as a novel approach to targeting tumour therapy.

**DOI:** 10.1038/bjc.1982.16

**Published:** 1982-01

**Authors:** J. Denekamp


Br. J. Cancer (1982) 45, 136

### Short Communication

### ENDOTHELIAL CELL PROLIFERATION AS A NOVEL APPROACH TO TARGETING TUMOUR THERAPY

J. DENEKAMP

*From the Gray Laboratory of the Cancer Research Campaign, Mount Vernon Hospital, Northwood, Middlesex HA6 2RN*

Received 20 July 1981 Accepted 7 October 1981

The proliferation rate of vascular endothelial cells in tumours exceeds that in most adult normal tissues by a very large factor, often greater than 20 (Figure). This enormous differential offers a potential route for aiming tumour therapy at solid tumours by means of a targeted systemic toxin, with little risk of damage to most of the normal tissues.

It has long been recognized that one of the major differences between solid tumours and adult normal tissues is the pattern and rate of development of the vascular network. New vessel formation in tumours is rapid, but it is insufficient, particularly in a 3-dimensional arrangement, to provide an adequate nutrient supply to all the tumour cells (Hirst *et al.*, 1982). For this reason many tumour cells are non-proliferating because of nutrient deprivation, and hypoxic because of oxygen depletion by the metabolizing tumour cells around each capillary. This produces resistance to both chemotherapy and radiotherapy. A great deal of research is devoted to finding ways of improving the oxygenation, proliferative status and drug delivery in such tumours.

Folkman and co-workers (Folkman *et al.*, 1971; Folkman, 1974, 1975) have recognized the special ability of tumour cells to promote new vessel formation via the tumour angiogenesis factor (TAF). They have proposed methods of preventing tumour growth by interfering with angiogenesis, including an immunological technique for inactivating TAF by producing an anti-TAF antibody which would prevent further capillary proliferation.

Elsewhere, little attention has been paid to the enormous *differential* between the proliferation characteristics of the tumour vasculature and the normal tissue vasculature. Tannock (1970), Hirst & Denekamp (1979) and Hirst *et al.* (1982) have compared the proliferation rate of the capillary endothelium in tumours with that of the tumour cells themselves. They concluded that the rate of endothelial proliferation limits tumour cell production even though as many as 18% of the endothelial cells can be in DNA synthesis at any one time.

Other studies have concentrated on the very low proliferation rate of endothelial cells in a variety of normal tissues (see reviews by Tannock & Hayashi, 1972; Hirst *et al.*, 1980). However, the remarkable difference in proliferation rates between endothelium in tumours and in normal tissues does not seem to have been previously commented upon or identified as a potential route for *directing* therapy at a tumour.

The Table summarizes the published labelling index (LI) for normal tissue and tumour endothelium, and illustrates the large difference between them. The values shown were obtained by scoring autoradiographs of tissues sampled $\frac{1}{2}$ h–1 h after a single injection of the DNA precursor, [$^3$H]dT. A mean LI of 0·6% is obtained for normal tissue endothelium, by averaging all the values reviewed by

TABLE.—*Proliferation of vascular endothelium in tumours and normal tissues*

| Normal tissues | LI (%) | Potential turnover time (Days) | Volume doubling time (Days) | Reference |
|---|---|---|---|---|
| Aorta (Mean of 7 studies) | 0·61 | 55 | ∞ | Reviewed by Tannock & Hayashi, 1972; Hirst *et al.*, 1980 |
| Arteries & Veins (Mean of 6 studies) | 0·82 | 41 | ∞ | |
| Capillaries (Mean of 14 studies) | 0·56 | 60 | ∞ | |
| Tumours | | (Hours) | | |
| C3H mammary carcinoma | 11·4 | 55 | 2·5 | Tannock, 1970 |
| C3H carcinoma KHH | 17·7 | 45 | 5 | Hirst *et al.*, 1982 |
| C3H carcinoma KHU | 17·9 | 45 | 6 | |
| WHT carcinoma RH | 4·5 | 178 | 13 | |

The potential turnover time is calculated as λTs/LI (assuming λ = 0·8 and Ts = 10 h) where λ is a correction factor for the non-linear distribution of cells throughout the phases of the cell cycle (Steel, 1968).

Tannock & Hayashi (1972) and Hirst *et al.* (1980) for a wide range of normal tissues. It does not differ much between major vessels and capillaries. Most authors have indicated extremely low proliferative activity in normal tissue endothelium, with half the studies showing an LI < 0.25%. The highest recorded value is 3·4% for adult rat mesenteric vessels (Crane & Dutta, 1964) but this contrasts markedly with 0·45% for the same tissue in mice (Hirst *et al.*, 1980).

Fewer data have been published for tumours; these are also summarized in the Table. The lowest LI is 4·5% for the exceptionally slow-growing mouse tumour CA RH (Hirst *et al.*, 1982) and two of the tumours have values approaching 18%. This difference between tumours and normal tissues is illustrated even more dramatically in the Figure. Some additional data are included for rat and mouse tumours (Denekamp & Hobson, in preparation) which show a range from 3·5% for lymphoma KHAA (which grows by infiltration rather than by evoking a neovasculature) to 32·5% for the rapidly growing rat fibrosarcoma $RIB_5$. The mean of the endothelial LI values in tumours is more than 20-fold that for normal tissues and there is hardly any overlap of the histograms.

With the advent of sophisticated immunological techniques, including monoclonal antibodies, it seems likely that this tremendous proliferative differential could be exploited. An immuno-histochemical technique is already in use to identify endothelial cells in histological preparations, by means of Factor VIII antigen (Hoyer *et al.*, 1973) and monoclonal antibodies have recently been raised against this antigen (Sola *et al.*, 1981). It should be possible to conjugate an S-phase-specific chemotherapeutic agent with an endothelial-cell-specific antibody, which could result in a large

FIGURE.—Vascular endothelial cell proliferation. A histogram showing the number of studies in normal tissues and in tumours with particular values for the labelling index of endothelial cells ½–1 h after a single injection of [³H]dT. Half of the normal-tissue studies ⊟ show values below 0·25% with a mean LI of 0·6%. The range for tumours ■ is from 3·5–32·5% with a mean of 13·5%. There is a very large differential in these LI values with almost no overlap.

degree of cell killing in tumour capillaries with very little damage to any normal tissue vessels. It is also conceivable that antibodies could be raised more specifically against proliferating endothelial cells, as distinct from all other endothelial cells. These could then be conjugated with any potent cell toxin (*e.g.* abrin or ricin) which would be released at the desired site of action. Proliferating endothelial cells can be produced very readily *in vivo* as a granulomatous response to an irritant; this would provide a richer and easier source of dividing endothelium than tumour vessels for raising antibodies This approach, directing tumour therapy via the proliferating endothelium, would avoid the necessity for identifying a tumour-specific antigen. It could provide a means of attacking solid tumours, including small metastases, via a universal pathway which is already known to be the weak point in tumour development. It would need to be used in combination with radiation and/or chemotherapy, and the sequencing would be very important. As vessels become occluded or collapse the surrounding cells would become more deficient in nutrients and oxygen and hence more chemo- and radiation-resistant. The possible influx of endothelial cells from the general circulation would also need to be studied, but this seems to be a relatively slow process (Hobson *et al.*, 1980).

Potential side effects, of course, would need a very thorough investigation in animals. Fresh wound tissue, premenopausal endometrium and placenta, are all tissues in which a highly proliferative endothelium will exist, and these would probably be at risk. Patients undergoing surgery, pregnant women, or those in the initial proliferative phase of each oestrus cycle, would therefore be unsuitable for this form of therapy. However, antiproliferating-endothelium therapy (APET) would be likely to be of only a few days duration to injure most of the tumour endothelium, as judged from the potential turnover-time estimates for tumour endothelium, at least for rodent tumours (Table). The demonstration of equally high LIs for endothelium in human tumours would of course be crucial for this approach to have a clinical future. This information should be readily obtainable with biopsy material and *in vitro* incubation (Denekamp & Kallman, 1973).

A small loss of endothelial cells in normal tissues is unlikely to be of major significance. Radiation studies on mesenteric arterioles have shown that the endothelium can be gradually depleted to 10–20% of its normal cell number without any thrombosis or failure of vessel function. (Hirst *et al.*, 1980). This functional reserve probably results from the well-developed sub-endothelial layers in normal blood vessels, which are notably lacking in most tumour vessels. It is not known how catastrophic the loss of 50% or 90% of the *tumour* endothelial cells would be, but it is possible that the already poor nturient supply would collapse and lead to massive tumour-cell necrosis.

We are currently studying a variety of other tumours and normal tissues. We are using repeated doses of [$^{3}$H]dT to determine how many sequential injections are needed to label all the endothelial cells. Preliminary data indicate that the 20-fold difference between tumour and normal vessels persists after one week of continuous labelling (Denekamp & Hobson unpublished). From these data it should be possible to predict how long an APET treatment with an S-phase-specific endothelial toxin would be needed to affect a given proportion of the endothelial cells in tumour capillaries.

I am very grateful to Mrs B. Hobson for her excellent assistance and to the Cancer Research Campaign for their financial support.

### REFERENCES

CRANE W. A. J. & DUTTA L. P. (1964) The influence of age on the uptake of $^{35}$S-Sulphate and $^{3}$H-Thymidine by the mesenteric arteries of rats with regenerating adrenal glands. *J. Pathol. Bacterial.* **88**, 291.

DENEKAMP J. & KALLMAN R. F. (1973) *In vitro* and *in vivo* labelling of animal tumours with tritiated thymidine. *Cell Tissue Kinet.*, **6**, 217.

FOLKMAN, J. (1974) Tumor angiogenesis factor. *Cancer Res.*, **34**, 2109.

FOLKMAN, J. (1975) Tumor angiogenesis. In *Cancer: A Comprehensive Treatise*, Vol. 3. (Ed. Becker). New York: Plenum. p. 355.

FOLKMAN, J., MERLER, E., ABERNATHY, C. & WILLIAMS, G. (1971) Isolation of a tumor factor responsible for angiogenesis. *J. Exp. Med.*, **133**, 275.

HIRST, D. G. & DENEKAMP, J. (1979) Tumour cell proliferation in relation to the vasculature. *Cell Tissue Kinet.*, **12**, 31.

HIRST, D. G., DENEKAMP, J. & HOBSON, B. (1980) Proliferation studies of the endothelial and smooth muscle cells of the mouse mesentery after irradiation. *Cell Tissue Kinet.*, **13**, 91.

HIRST, D. G., DENEKAMP, J. & HOBSON, B. (1982) Proliferation kinetics of endothelial cells and tumour cells in three mouse mammary carcinomas. *Cell Tissue Kinet.*, (In press).

HOBSON, B., HIRST, D. G. & DENEKAMP, J. (1980) Endothelial Cell Proliferation in Tumours. *Gray Lab. Ann. Rep.*, **11**, 47.

HOYER, L. W., DE LOS SANTOS, R. P. & HOYER, J. R. (1973) Antihemophilic factor antigen: Localisation in endothelial cells by immunofluorescent microscopy. *J. Clin. Invest.*, **52**, 2737.

SOLA, B., AVNER, P., SULTAN, Y., JEANNEAU, C. & MAISONNEUVE, P. (1981) Monoclonal antibodies against the human factor VIII/von Willebrand factor molecule inhibiting antihemophilic factor and ristocetin cofactor activities. *C. R. Acad. Sci.* III, **292**, 1055.

STEEL, G. G. (1968) Cell loss from experimental tumours. *Cell Tissue Kinet.*, **1**, 193.

TANNOCK, I. F. (1970) Population kinetics of carcinoma cells, capillary endothelial cells and fibroblasts in a transplanted mouse mammary tumour. *Cancer Res.*, **30**, 2470.

TANNOCK, I. F. & HAYASHI, S. (1972) The proliferation of capillary endothelial cells. *Cancer Res.*, **32**, 77.